# Supplementary material for: Three-dimensional hierarchically porous MoS2 foam as high-rate and stable lithium-ion battery anode
Source: Nat Commun. 2022 Oct 12;13:6006. doi: 10.1038/s41467-022-33790-z (PMC9556660; doi:10.1038/s41467-022-33790-z)
Supplement: Supplementary file 1 — Supplementary Information [file 41467_2022_33790_MOESM1_ESM.pdf]

Three-dimensional Hierarchically Porous MoS_2 Foam as High-Rate and Stable Lithium-ion Battery Anode

Xuan Wei[1], Chia-Ching Lin[2], Chuanwan Wu[3], Nadeem Qaiser[1], Yichen Cai[1], Ang-Yu Lu[4], Kai Qi[1], Jui-Han Fu[5], Yu-Hsiang Chiang[1], Zheng Yang[1], Lianhui Ding[6], Ola. S. Ali[6], Wei Xu[6], Wenli Zhang[7], Mohamed Ben Hassine[1], Jing Kong[4], Han-Yi Chen[2], and Vincent Tung[1,5]**

[1]Physical Science and Engineering Division, King Abdullah University of Science and Technology, Thuwal, 23955-6900, Saudi Arabia
[2]Department of Materials Science and Engineering, National Tsing Hua University, Hsinchu, 300 Taiwan
[3]Molecular Foundry, Lawrence Berkeley National Lab, Berkeley, California 94720, USA
[4]Department of Electrical Engineering, Massachusetts Institute of Technology, Cambridge, Massachusetts 02139, USA
[5]Department of Chemical System Engineering, School of Engineering, The University of Tokyo, Tokyo 113-8656, Japan
[6]Saudi Aramco, Chemicals R&D Lab at KAUST, Research and Development Center, Thuwal, 23955-6900, Saudi Arabia
[7]Guangdong Provincial Key Laboratory of Plant Resources Biorefinery, School of Chemical Engineering and Light Industry, Guangdong University of Technology (GDUT), 100 Waihuan Xi Road, Panyu District, Guangzhou 510006, China

*To whom correspondence should be addressed: hanyi.chen@mx.nthu.edu.tw; and vincent@g.ecc.u-tokyo.ac.jp

Supplementary Note

EHD condition optimization. Under different combinations of flow rate, charge (Q), and electric field strength (E), droplets are jetted in different modes. When E and Q are low, the large droplets are jetted out from the nozzle mainly due to gravity in the "dripping mode." Increasing the flow rate renders fluid to form a jet or stream, namely "jet mode." In both dripping and jet modes, the large droplets or streams coalesce into thick films on the targeted substrate. On the other hand, the "cone-jet mode" emerges when E exceeds the threshold. The cone-jet mode is characterized by the formation of the Taylor-cone-shaped extrusion near the tip of the injection needle. Usually, a higher flow rate requires a higher voltage to reach a cone-jet mode. However, tilted jets or multiple jets may occur when E is too high. The result is the over-dispersed tiny droplets, but the spray is not stable and less controllable. Particularly, the "cone-jet mode" enables the controllability and stability of extruding the ce-MoS_2 suspension into self-dispersing and electrostatically charged droplets with a very narrow distribution in diameter (diameter is about 150 nm) blended with continuous jet streams.

The stable generation of cone-jet mode is critical for forming ordered 2D patterns and thus the final 3D hierarchical architecture. The optimized conditions for MoS_2 foam formation are of 0.75 kV/cm (E), 7 µL/min (flow rate) and 200°C (substrate temperature). **Supplementary Figure 1** schematically illustrated the operational conditions and the formation of different structures. For control experiments, the flow rate and temperature were 20 µL/min and room temperature 25°C for MoS_2 wrinkled films and 5 µL/min and 200°C for crumples, respectively, while other conditions were all the same. MoS_2 bulk anodes were prepared by drop-casting the 200 µL powder MoS_2 solution (5 mg/ml, the solvent is DI-H_2O) on Cu foil, then annealing at 200°C for 3 hours.

Diffusion Coefficient

The diffusion coefficient could be derived from Fick's second law, based on the long-time approximation, as shown in the following equation:

$$D_{Li^+} = \frac{dln(I)}{dt} \cdot \frac{4L^2}{\pi^2} \quad \text{(Supplementary Equation 1)}$$

Where I is the step current, and t is the step time. L is the diffusion distance, calculated using the relationship.

$$L = V_M * n_B / S$$ (Supplementary Equation 2)

Where V_M is 31.63438735 cm^3 mol^{-1}, n_B is 6.25 x 10^{-6} mol and S is 1.13 cm^2.

Supplementary Information

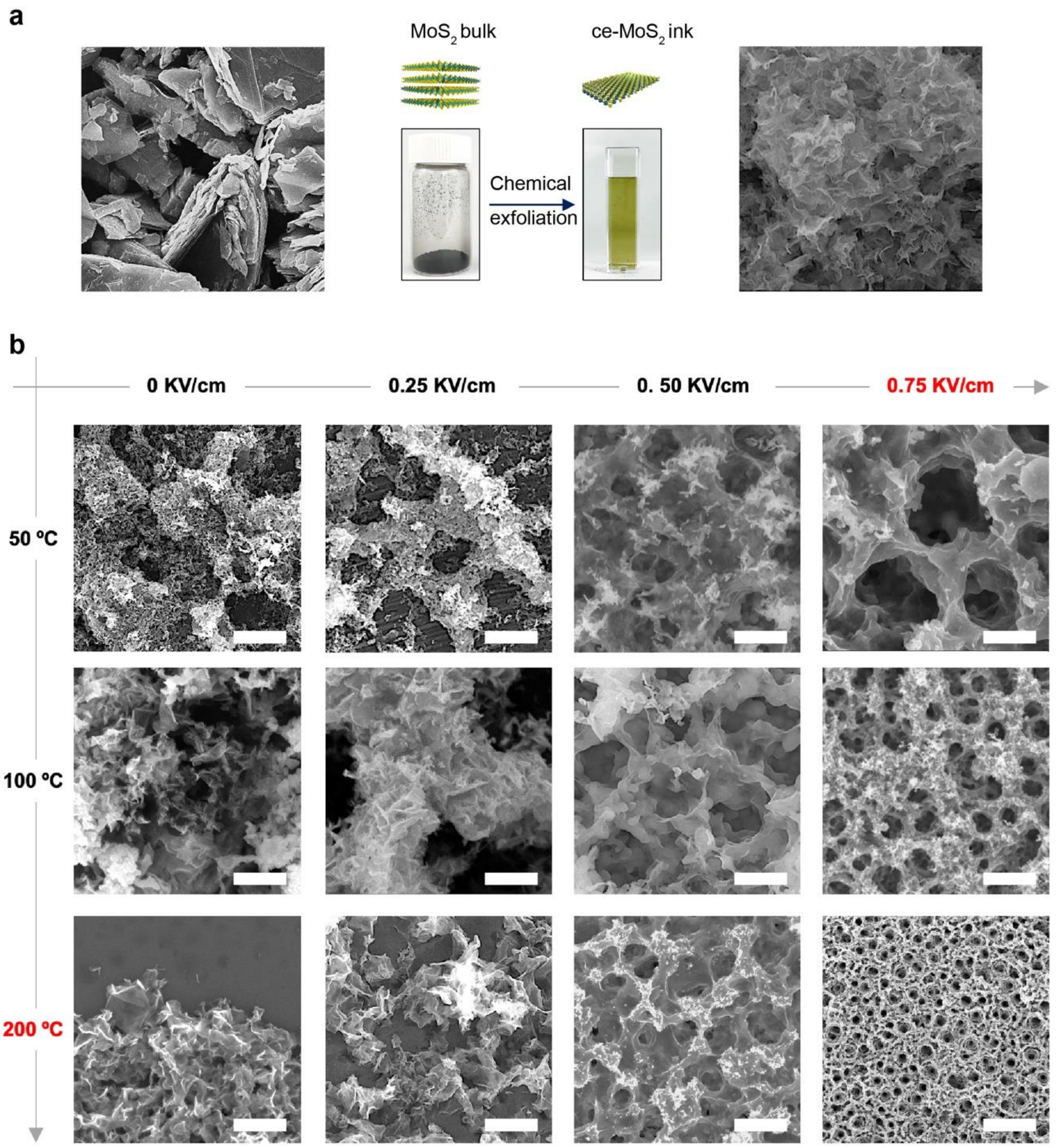

Supplementary Figure 1 | Schematic illustrations of different EHD modes and the resultant morphologies. (a) Commercially available MoS_2 bulk powder and ce-MoS_2 aqueous dispersion and their corresponding SEM images. (b) Arrays of SEM images were taken under a different combination of annealing temperature and electric field. MoS_2 wrinkled films formed under the jet stream mode while MoS_2 foam under the cone-jet (jet stream and droplets) mode. MoS_2 crumples under high temperature and electric field. Scale bars from left to right, 200 nm, 200 nm, 1 µm, and 2 µm, respectively.

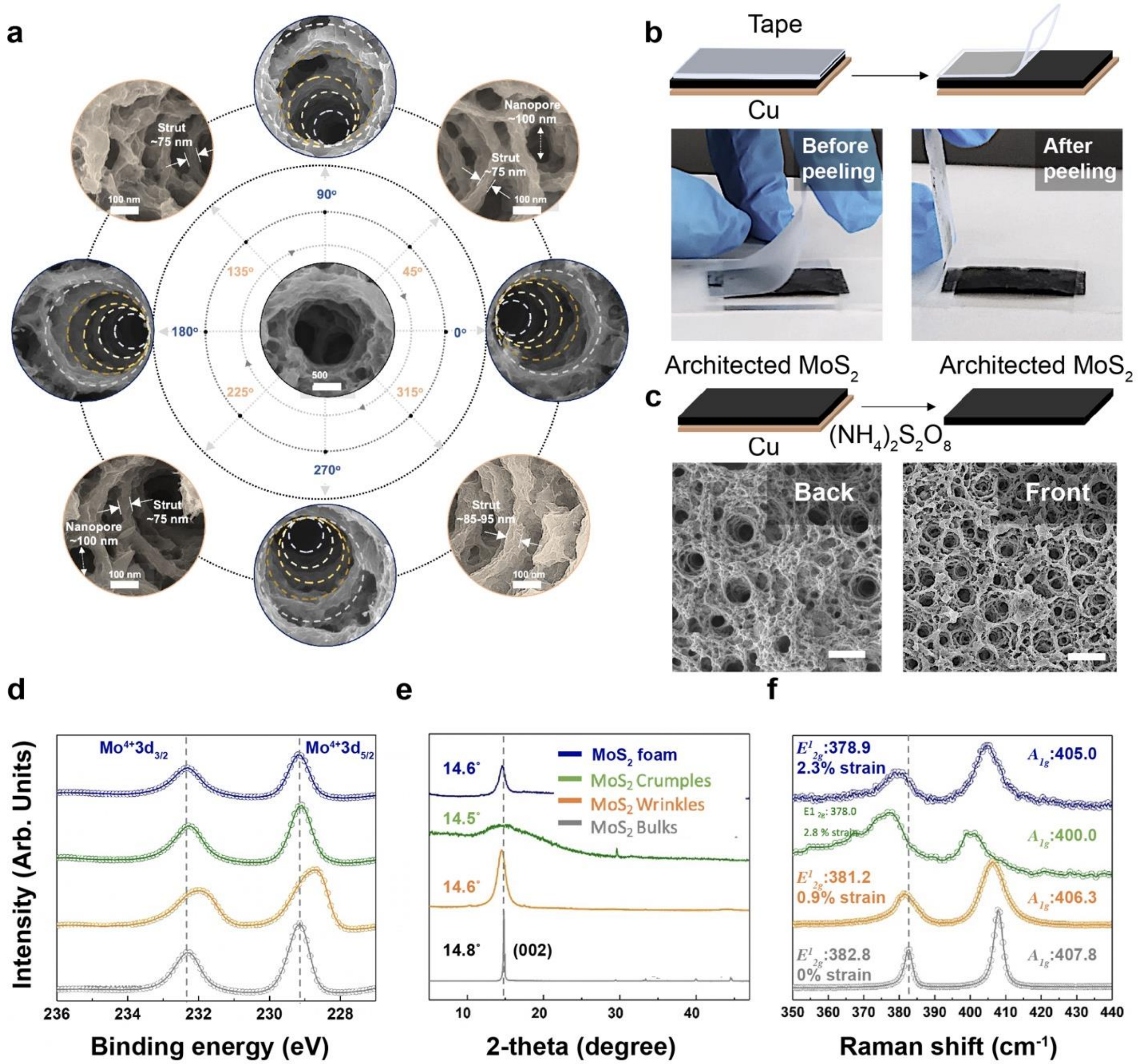

Supplementary Figure 2 | Imaging, mechanical, and spectroscopic characterization of MoS_2 foam. (a) Reconstruction of MoS_2 foam SEM images taken at different perspectives (peripherals, tilted view) near the selected spot (center, top view) collectively attests to forming uniform, layered sponge-like nanostructures with interconnected struts and spatially distributed nanopores. Note that each layer is false-colored with dotted lines for clarity. Meanwhile, HRSEM images (false-colored in light orange) further reveal detailed nanoscale features that comprise thin, wrinkled struts with an average thickness of 75~95 nm and nanopores in oval shapes with a bimodal average distribution of diameters of 85 and 200 nm, respectively. (b) MoS_2 foam cannot be peeled off by tape and can only be removed by dissolving Cu substrate in ammonium persulfate solution (c).

(d) High-resolution XPS spectra of Mo *3d* prove pure 2H phase in MoS_2 foam. Except wrinkled films is in the 1T phase (because it formed by the restacking of ce-MoS_2 under 25°C), the other three samples are all in the 2H phase (unstable 1T phase changes into stable 2H phase at 200°C). No permanent changes in S are observed during the structural deformation. (e) XRD spectra indicate that MoS_2 foam exhibits more expansive interlayer space and higher irregularity than bulk. (f) Raman spectra show a significant shift in the E^1_{2g} peak in MoS_2 foam, indicating the high strain load on the overall nanostructure. The vertical black dash lines indicate peak positions from the MoS_2 standard.

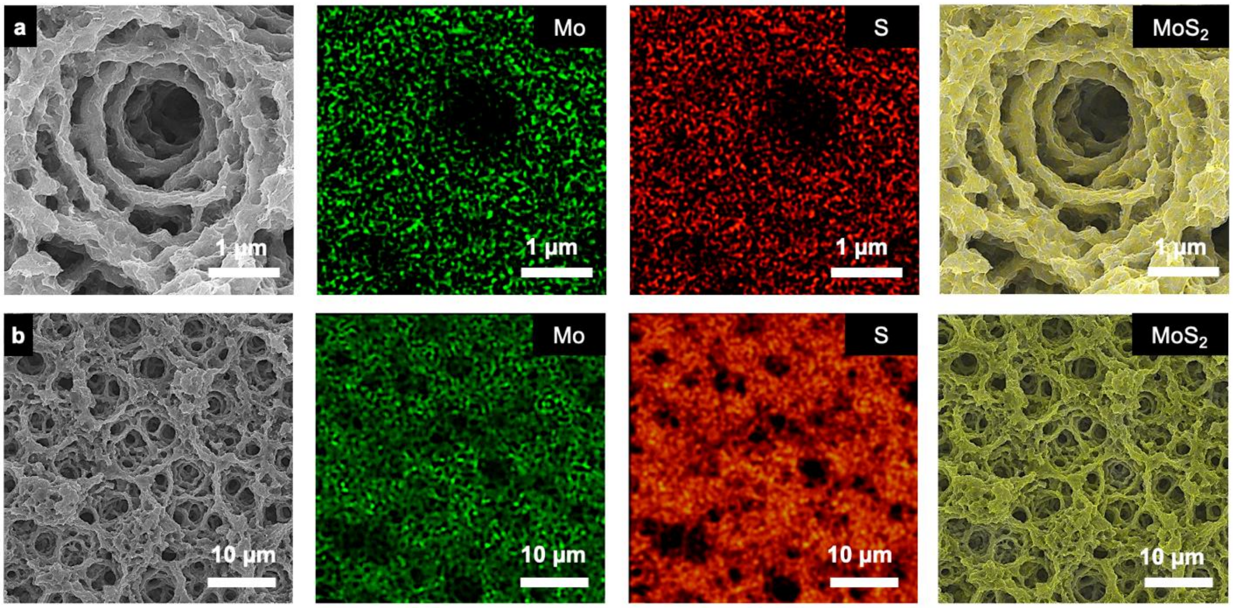

Supplementary Figure 3 | Chemical coherence of MoS_2 foam. Energy-dispersive X-ray spectroscopy (EDX) mapping of relevant elements in (a) a single truss unit and (b) within percolated networks, including Mo in green and S in orange, confirms the chemical coherence across the entire architected MoS_2.

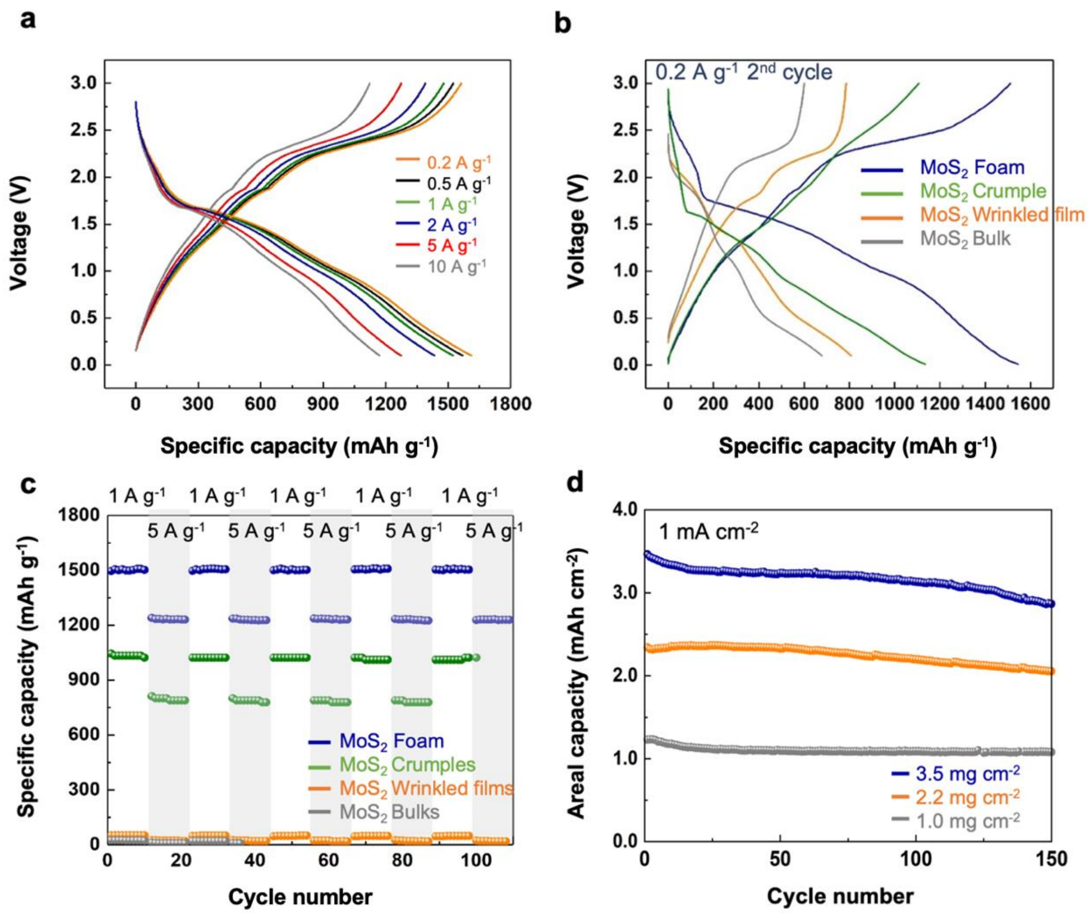

Supplementary Figure 4 | Electrochemical properties of 3D MoS_2 foam anode. (a) Galvanostatic discharge and charge profiles of MoS_2 foam were measured at different current densities. Multiple sequential plateaus exist in the charging and discharging process, indicating continuous multi-step lithiation and delithiation. (b) The 2nd cycle galvanostatic discharge and charge profiles at the current density of 0.2 A g^{-1} comparisons of MoS_2 foam with reference samples show different plateaus distribution. (c) Dynamic cycling performances at alternating current densities of 1A g^{-1} and 5 A g^{-1} for every ten cycles. (d) High aerial mass loading test (up to 3.5 mg cm^{-2} active material) of MoS_2 foam anodes.

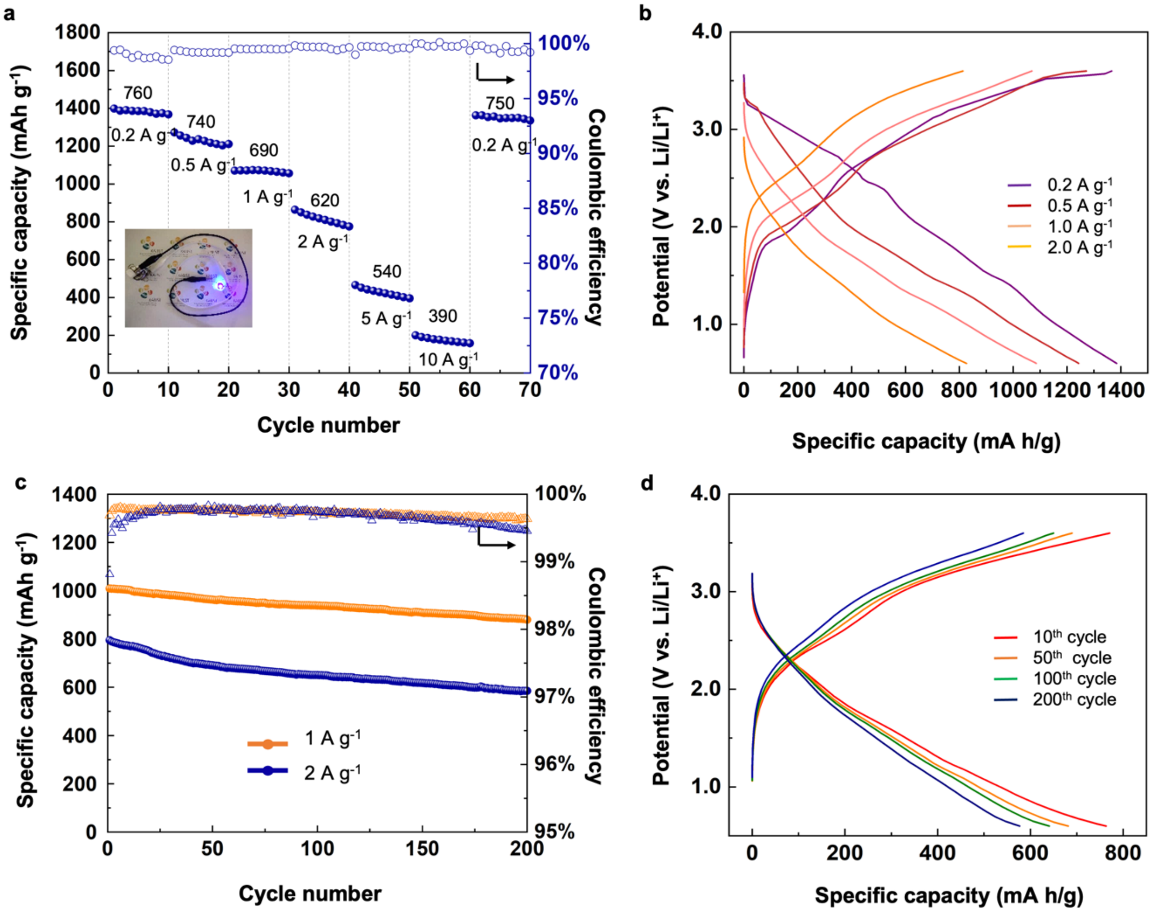

Supplementary Figure 5 | Electrochemical performance of MoS_2 foam // $LiFePO_4$ full cell. (a) Rate performance. (b) Voltage profiles plotted as a function of the current density in the voltage range of 0.6-3.6 V. (c) Specific capacity and Coulombic efficiency measured at the current density of 1 and 2 A g^{-1}, respectively. The specific capacity of the full cell is calculated based on the mass of the MoS_2 anode electrode. (d) Voltage profiles are plotted as a function of cycles at a discharge rate of 2 A g^{-1}.

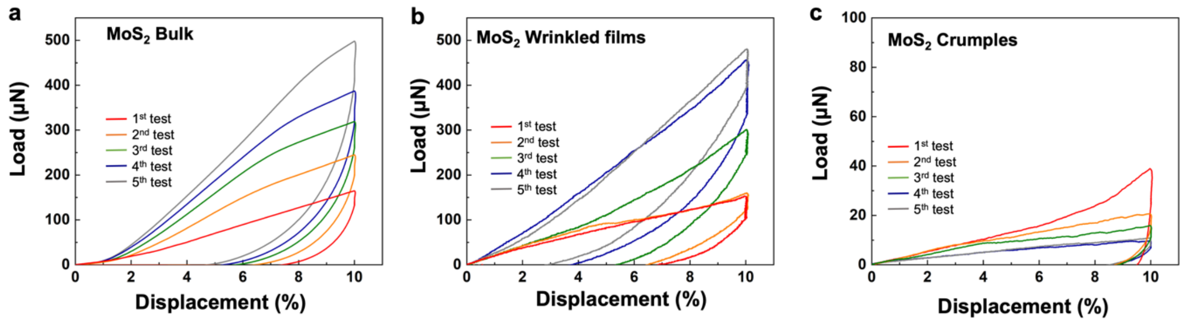

Supplementary Figure 6 | Mechanical measurement under fixed displacement (10%). (a) MoS_2 bulk and (b) wrinkled films show a similar response. After release, they can slowly recover from 10% to around 7-8%, along with a curve behavior in the first cycle. They become more and more compressed in the following cycles, showing a strain-hardening effect. (c) Crumples have the weakest mechanical strength. It completely collapses after the first cycle.

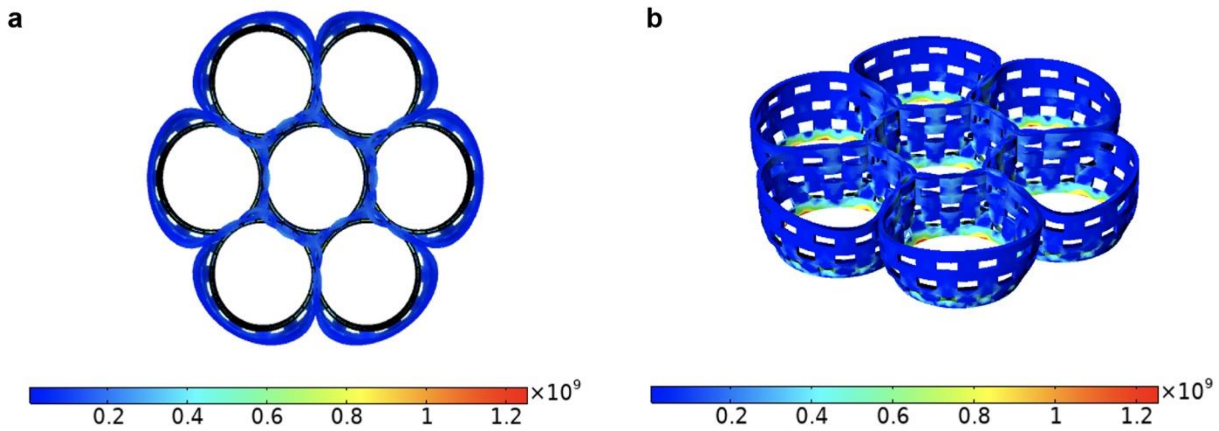

Supplementary Figure 7 | Von Mises stress distribution simulated at a SOC of 100%. (a) Top and (b) perspective views of the 3D architected MoS_2 foam confirm that the structural integrity is preserved and shows no sign of electrochemomechanical fatigue and fracture.

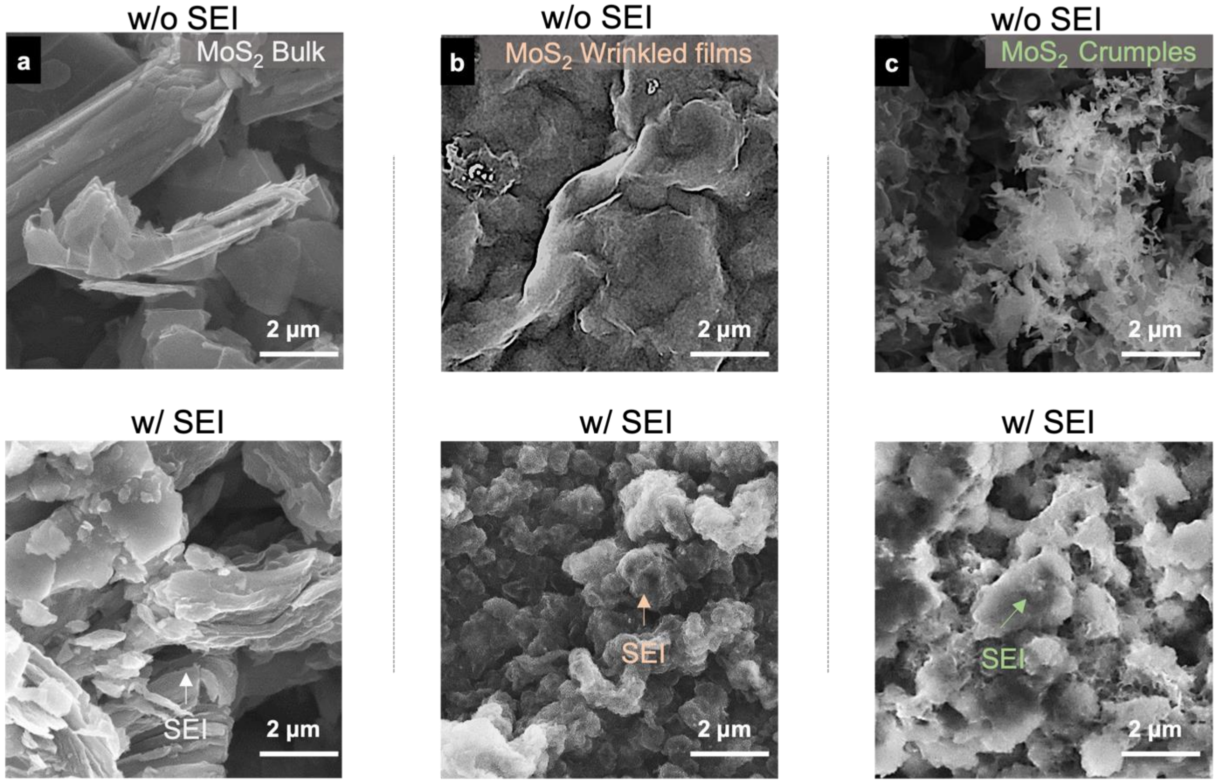

Supplementary Figure 8 | SEM images of MoS_2 reference electrodes before (top) and after (bottom) the deposition of secondary electrolyte interphase (SEI) (1000 cycles). (a) MoS_2 bulk, (b) wrinkled films, and (c) crumples display rampant growth of SEI and thus the congested ion transport channels.

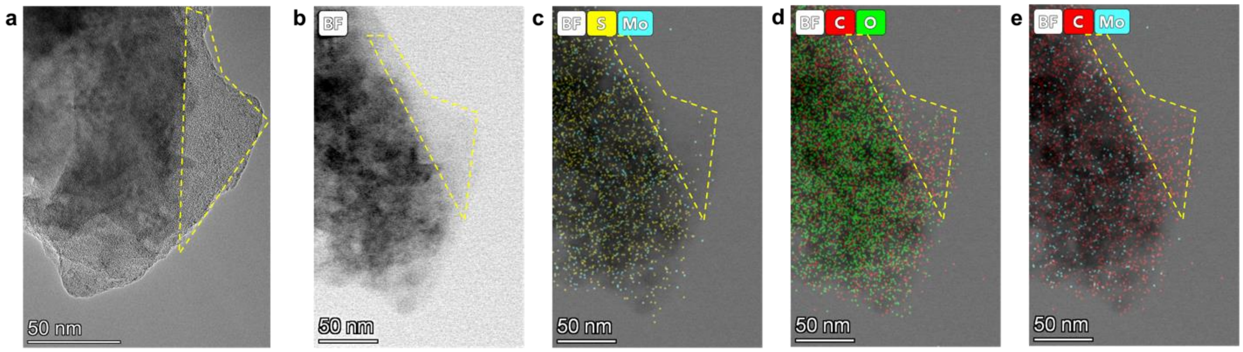

Supplementary Figure 9 | Formation of secondary electrolyte interphase (SEI) layer after post-cycling of MoS_2 foam. (a) TEM, (b) STEM images, and (c-e) EDX mappings, where S is in yellow, Mo is in cyan, C is in red, and O is in green, reveal the spatial and uniform deposition of SEI on MoS_2 foam. The boundary between MoS_2 and SEI layer (represented by C in red and O in green) can be determined by the corresponding EDX mapping and compositional analysis in Supplementary Table 4.

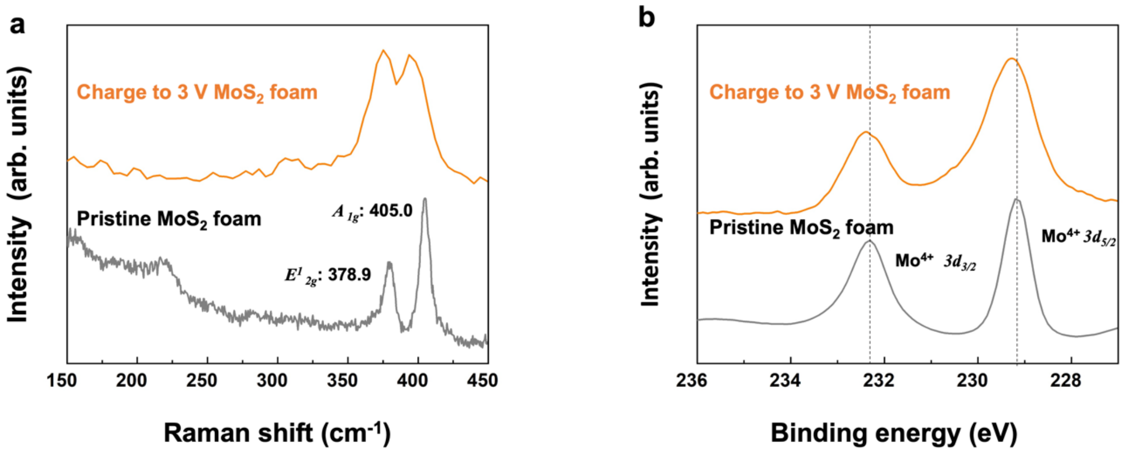

Supplementary Figure 10 | Post-cycling characterization of MoS_2 foam. (a) Raman and (b) X-ray photoelectron spectroscopy (XPS) spectra of pristine MoS_2 foam (color in gray) and MoS_2 foam after scanning for 1000 cycles (color in orange).

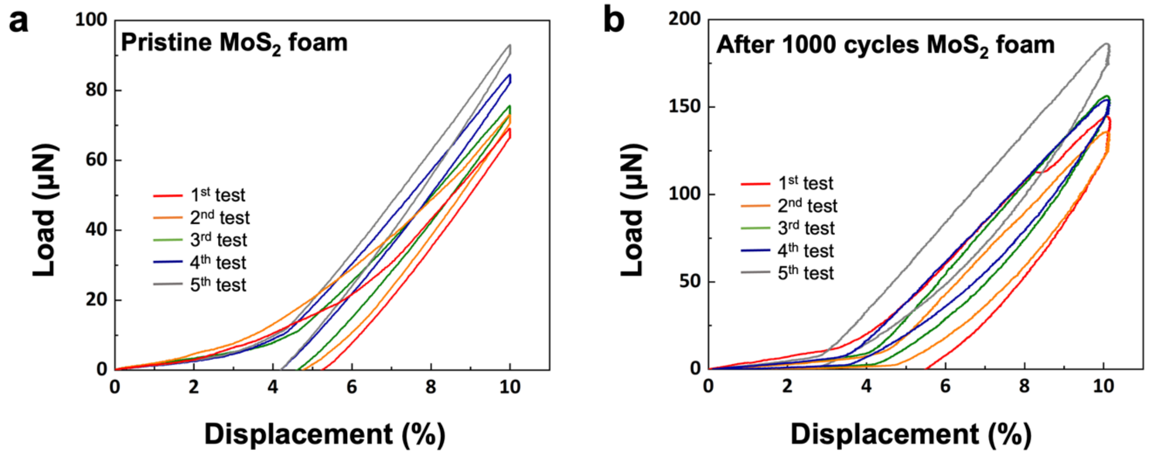

Supplementary Figure 11 | Absence of brittle behavior. Mechanical test of MoS_2 foam (a) before and (b) after post-cycling for 1000 cycles.

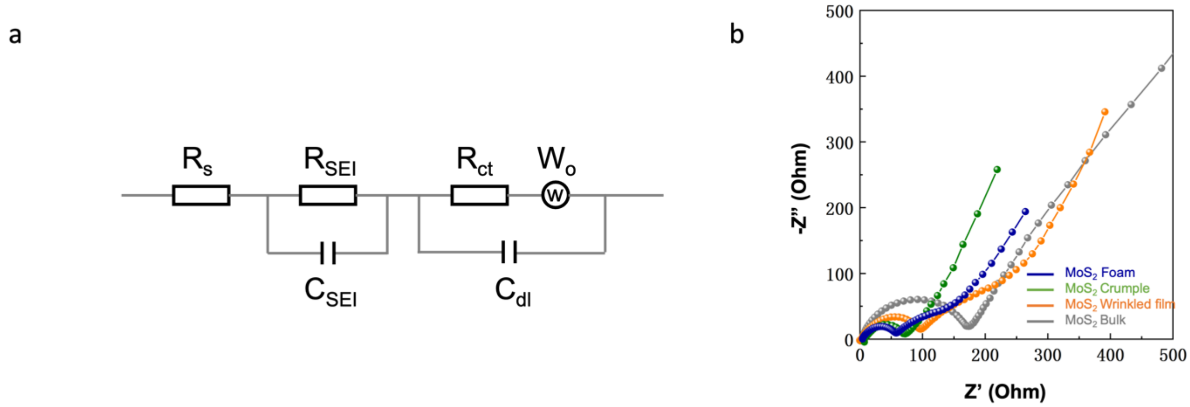

Supplementary Figure 12 | EIS analysis of MoS_2 bulk, wrinkled films, crumples, and foam. (a) The equivalent circuit model of MoS_2 foam electrode. (b) Nyquist plots of MoS_2 anodes were taken at a fully discharged state after 10 cycles and at 100 mA g^{-1}.

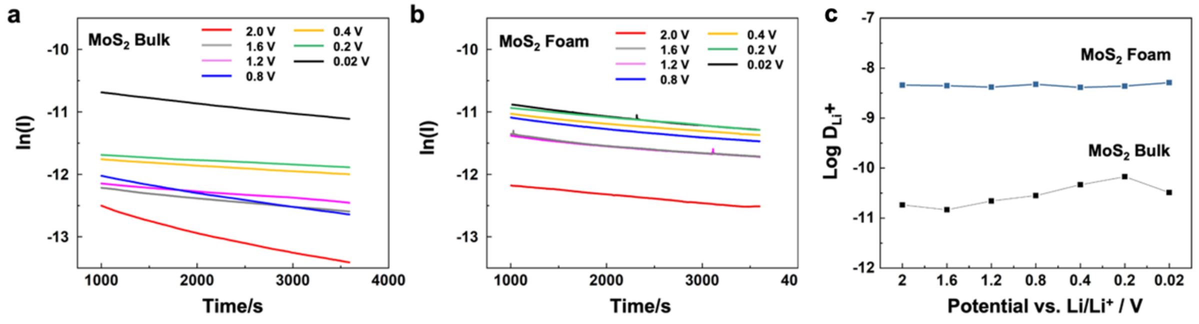

Supplementary Figure 13 | Calculating diffusion coefficient by a potentiostat intermittent titration technique (PITT). $ln(I)$ vs. time in (a) MoS_2 bulk and (b) MoS_2 foam as an anode for LIBs. (c) Diffusion coefficient values (D_{Li+}) in terms of logarithms to the base 10 of MoS_2 foam and MoS_2 bulk.

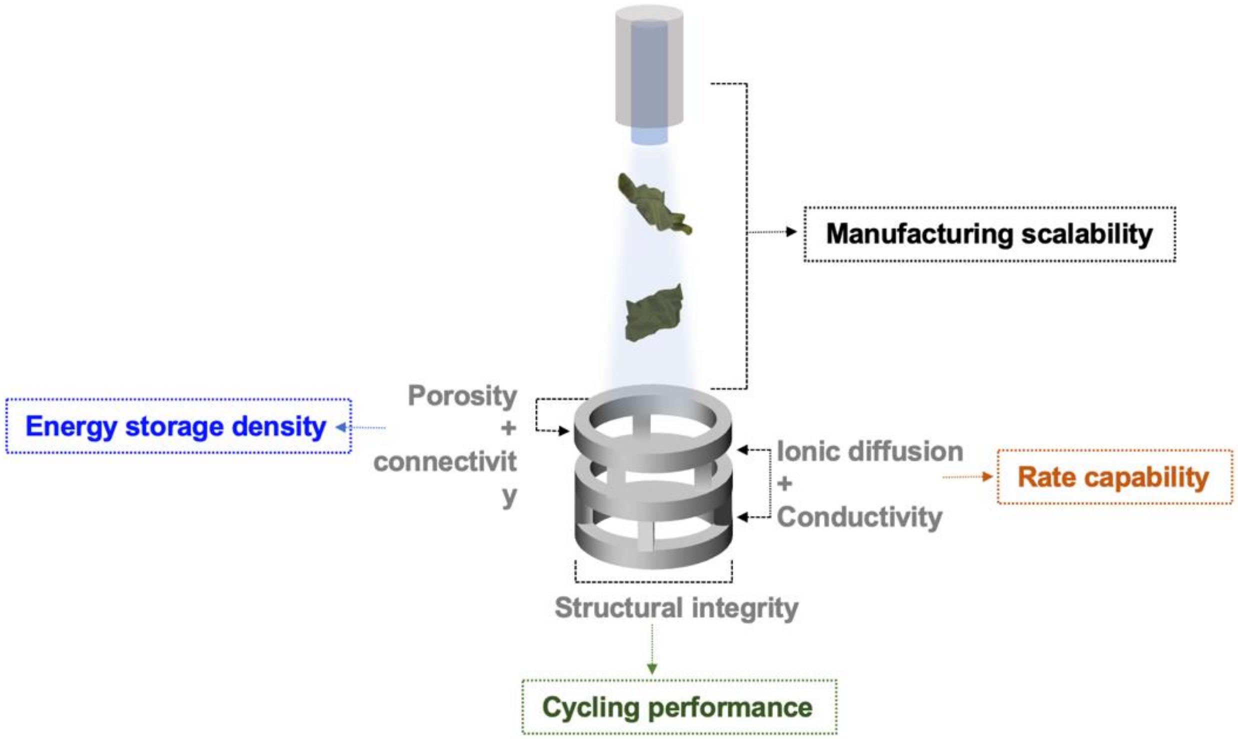

Supplementary Figure 14 | Simultaneously enhanced electrochemical characteristics through evaporation induced printing. A schematic illustration that summarizes the figures of merit of EHD-printed 3D architected MoS_2 foam.

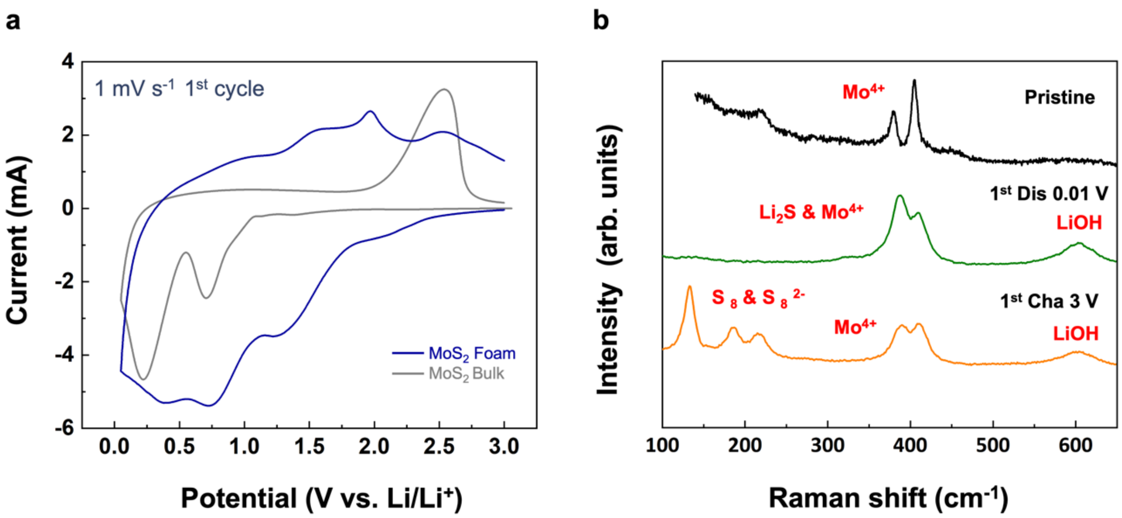

Supplementary Figure 15 | Electrochemical reaction of MoS_2 foam during the 1st cycle. (a) The 1st cycle CV profiles of MoS_2 foam and MoS_2 bulk electrode measured at 1 mV s^{-1} in the voltage window of 0.01–3 V. (b) Ex-situ Raman comparison of pristine, 1st discharge, and 1st charge of MoS_2 foam.

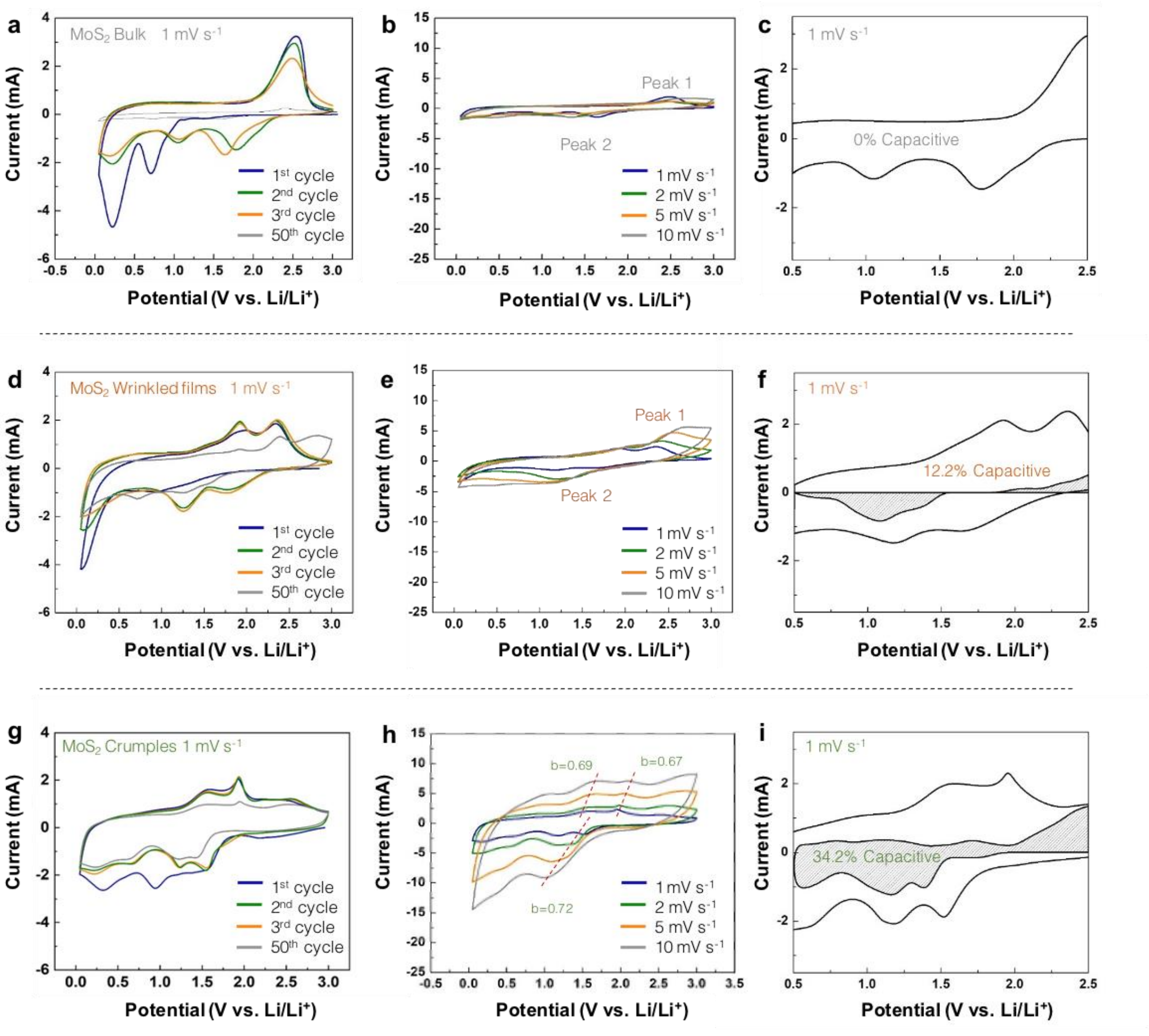

Supplementary Figure 16 | Pseudocapacitive energy storage ratios to the total charge storage in reference samples. (a, d, and g) The 1st, 2nd, 3^{rd,} and 50th cycle CV curves were collected at 1mV s^{-1}. (b, e, and h) The 3rd cycle CV curves collected at 1, 2, 5, 10 mV s^{-1}. (c), (f) and (i) Capacitive charge storage contributions cycled at 1 mV s^{-1} are denoted by the shaded grey region.

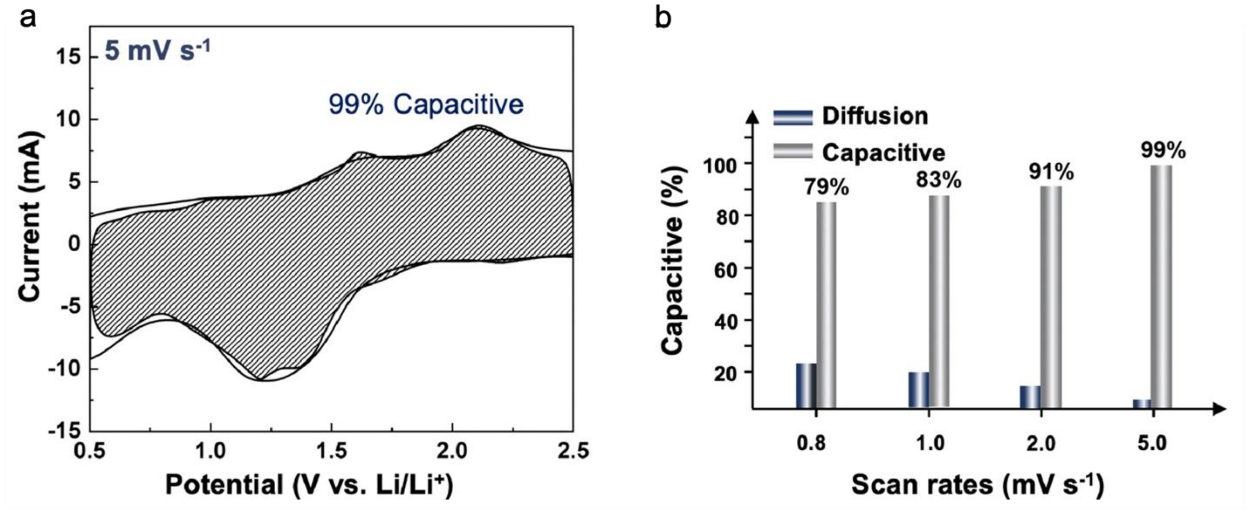

Supplementary Figure 17 | Predominant capacitive behavior derived from the hierarchically porous MoS_2. (a) Capacitive and diffusion-controlled charge storage contributions for MoS_2 foam cycled in a Li-ion electrolyte at a scan rate of 5 mV s^{-1}. (b) The bar chart showcases the capacitive contribution increases at the high scan rates.

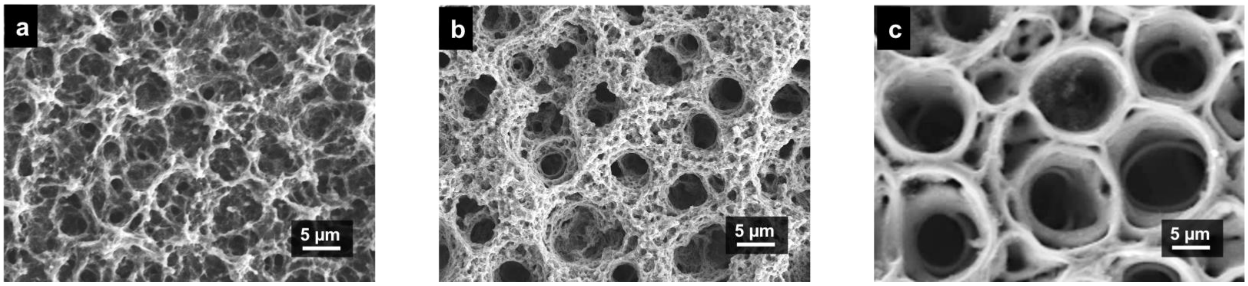

Supplementary Figure 18 | Comparison of different 2D materials in foam-like hierarchical structures fabricated through dewetting-induced manufacturing. SEM images of (a) reduced graphene oxide (rGO) foam (b) MoS_2 foam and (c) $Ti_3C_2T_x$ MXene foam. Because of each precursor's different thickness and mechanical properties, the distribution of pore size and structure hierarchies vary significantly.

	Rate Capability (mA h g^{-1})						Capacity Retention			Synthetic procedure		Reference
	0.2 A g^{-1}	0.5 A g^{-1}	1 A g^{-1}	2 A g^{-1}	5 A g^{-1}	10 A g^{-1}	Rev. capacity (mA h g^{-1})	cycles	current density (A g^{-1})	Process and temperature	# of Steps	
MoS_2 Foam	1575	1550	1515	1431	1268	1111	750<br>450	1000<br>1000	5<br>10	185 ℃	1	This work
MoS_2 nanosheets vertically grown on graphene	1035	986	890	N/A	N/A	N/A	907	400	1	Hydrothermal method and in-situ growth 800 ℃	5	1
Vertically aligned metallic MoS_2	1080	1048	1020	978	875	725	1150	350	5	Hydrothermal method 200 ℃	3	2
Column-like MoS_2 superstructure	930	N/A	700	N/A	390	200	650	500	1	Wet-chemical approach 220 ℃	2	3
MoS_2/SWNT composite	N/A	1146	N/A	N/A	975	710	950	500	2	Hydrothermal	3	4
Honeycomb-like MoS_2	1172	1095	1007	966	800	N/A	1100	60	0.2	200 ℃	3	5
MoS_2/CNT tubular structures	N/A	1115	1000	926	776	670	800	1000	5	Fabrication Annealing	6	6
MoS_2 hollow nanospheres	895	831	762	711	576	N/A	1100	100	5	Solvothermal	3	7

Supplementary Table 1 | Comparison of rate capability, cycling stability, and synthetic procedure of MoS_2 foam and the other recent MoS_2 composite anodes.[1–7]

Material	Current density (A g $^{-1}$)	Gravimetric capacity (mAh g $^{-1}$)	Packing density (g cm $^{-3}$)	Volumetric capacity (Ah L $^{-1}$)	Current density (mA cm $^{-2}$)	Areal Capacity (mAh cm $^{-2}$)	Ref.
MoS_2 Foam	1 (10 cycles)	1515	1	1515	1	1.515	This work
	5 (1000 cycles)	1092		1092	5	1.092	
	10 (1000 cycles)	773		773	10	0.773	
MoS_2/CNT tubular structures	5 (1000 cycles)	776	0.3	170	5.0	0.8	6
(BP-G)/PANI (10th cycle)	2.6 (2000 cycles)	730	1.5	1090	3.1	1.1	8
	5.2 (2000 cycles)	630		940	6.3	0.95	
	13 (2000 cycles)	350		530	15.6	0.53	
Si @ Graphene (325th cycle)	2.1	1160	0.65	750	1.7	1.2	9
$Nb_{18}W_{16}O_{93}$ (750th cycle)	3.0	100	1.8	200	7.5	0.35	10
Si/Graphite (100th cycle)	0.26	500	1.6	790	1.7	3.4	11

Supplementary Table 2 | Comparison of gravimetric and volumetric capacities between MoS_2 foam and other state-of-the-art anode materials after cycling at high current densities. Note that the gravimetric capacities are calculated from the total electrode material (e.g., MoS_2), which does not contain any additive or binder. The volumetric capacity results are computed from the gravimetric capacity and packing density of the anodes. [6,8–11]

Anode	Cathode	Mass ratio (Anode: Cathode)	Voltage range (V)	Working voltage (V)	Specific capacity (mA h g^{-1})	ED[a] (Wh/kg and PD[b] (W/kg)	Reference
**MoS_2 Foam**	LFP	1:10	0.6-3.6	2.2	1072 at 1 A g^{-1}	2723 and 2270 at 200 mA/g （anode mass) 248 and 207 at 200 mA/g (total mass)	**This work**
**Graphene nanoflakes**	LFP	--	1.0-4.0	3.0	165 at 1 C	190 and 196 at 1 C (total mass)	**12**
**N-PSi@C**	LCO	-	3.0-4.3	3.8	1.5 mAh cm^{-2}	1621 Wh/L with a $LiCoO_2$ cathode and a power density of 7762 W/L with a $LiFePO_4$ cathode. (anode mass)	**13**
**Si/CIWGS**	$Li(Ni_{0.75}Co_{0.1}Mn_{0.15})O_2$	--	2.7-4.2	3.6	196 at 1 C	240 and 245 at 200 mA/g (total mass)	**14**
**Yolk-shelled Si/C**	LFP	-	2.0-4.0	2.8	150 at 50 mA g^{-1}	--	**15**
**TiO_2-C**	LCO	--	2.0-4.75	3.75	240 at 0.2 A g^{-1}	413 and 100 at 100 mA/g (anode)	**16**
**TiO_2-MoO_3**	LCO	4:1	1.0-4.0	3.3	120 at 0.05 A g^{-1}	285 and 1086 at 50 mA/g (total mass)	**17**
**TiO_2 nanofiber**	LMO	1:1.9	1.7-2.5	2.2	105at 1 C	220 at 100 mA/g (cathode)	**18**
**Al yolk–shell nanoparticles**	LFP	1:7.7	2.5-4.0	3.3	1200 at 1C	--	**19**
**Ge nanorod**	LCO	--	2.8-3.9	3.3	1184 at 0.5 C	475 and 6587 at 5 C (anode)	**20**
**Sn-C**	C-LFP	1:2	1.8–3.5	2.8	120 at 0.5 C	340 and 2400 at 5 C (cathode mass)	**21**
**Si@C**	AC	1:6	2.0-4.0	3.0	68.3 at 5 C	189 and 10174 at 2.5A/g (total mass)	**22**

[a]ED: energy density; [b]PD: power density
Some are calculated by the anode or cathode mass while some are based on total mass of anode and cathode.

Supplementary Table 3 | Summary of electrochemical performance of full LIBs with different anode materials.[12–22]

Solid Electrolyte Interphase (SEI)					Electrodes	
Z	Element	Family	Atomic fraction (%)	Atomic error (%)	Atomic fraction (%)	Atomic error (%)
6	C	K	75.23	20.35	30.49	5.51
8	O	K	20.37	7.11	53.4	14.28
9	F	K	1.3	1.48	1.32	0.55
15	P	K	0.36	0.82	0.72	0.31
16	S	K	1.5	2.56	7.46	2.16
42	Mo	K	1.25	2.35	6.61	1.59

Supplementary Table 4 | SEI and electrode composition analyses derived from Supplementary Figure 9.

Fitting parameters	R_s	R_{SEI}	C_{SEI} (μF/cm²)	R_{ct}	C_{dl} (mF/cm²)	A (Ω S^{-n})	n
MoS_2 foam	3.93	51.56	27.76	89.49	4.29	0.58	0.29
MoS_2 crumples	7.37	72.61	110.78	44.78	5.51	12.88	0.35
MoS_2 wrinkled films	2.74	97.50	33.87	173.82	3.80	31.61	0.32
MoS_2 bulk	3.32	186.14	17.95	163.30	6.92	136.97	0.57

Supplementary Table 5 | Values of R_e, R_{SEI}, C_{SEI}, $R_{ct,}$ and C_{dl} are obtained by fitting data to Supplementary Figure 12.

Supplementary Reference:

1. Teng, Y. *et al.* MoS_2 Nanosheets Vertically Grown on Graphene Sheets for Lithium-Ion Battery Anodes. *ACS Nano* **10**, 9, 8526-8535 (2016).
2. Jiao, Y. *et al.* Ion Transport Nanotube Assembled with Vertically Aligned Metallic MoS_2 for High Rate Lithium-Ion Batteries. *Adv. Energy Mater.* **8**, 1–9 (2018).
3. Ding, J., Zhou, Y., Li, Y., Guo, S. & Huang, X. MoS_2 Nanosheet Assembling Superstructure with a Three-Dimensional Ion Accessible Site: A New Class of Bifunctional Materials for Batteries and Electrocatalysis. *Chem. Mater.* **28**, 2074–2080 (2016).
4. Liu, Y. *et al.* Electrical, Mechanical, and Capacity Percolation Leads to High-Performance MoS_2 / Nanotube Composite Lithium Ion Battery Electrodes. *ACS Nano* **10**, 6, 5980-5990 (2016).
5. Wang, J. *et al.* Self-Assembly of Honeycomb-like MoS_2 Nanoarchitectures Anchored into Graphene Foam for Enhanced Lithium-Ion Storage. *Adv. Mater.* **26**, 7162–7169 (2014).
6. Chen, Y. M., Yu, X. Y., Li, Z., Paik, U. & Lou, X. W. Hierarchical MoS_2 tubular structures internally wired by carbon nanotubes as a highly stable anode material for lithium-ion batteries. *Sci. Adv.* **2**, e1600021 (2016).
7. Wang, Y., Yu, L., Wen, X. & Lou, D. Synthesis of Highly Uniform Molybdenum – Glycerate Spheres and Their Conversion into Hierarchical MoS_2 Hollow Nanospheres for Lithium-Ion Batteries. *Angew. Chem. Int. Ed.* 7423–7426 (2016).
8. Jin, H. *et al.* Black phosphorus composites with engineered interfaces for high-rate high-capacity lithium storage. *Science* **370**, 192-197 (2020).
9. Li, Y. *et al.* Growth of conformal graphene cages on micrometer-sized silicon particles as stable battery anodes. *Nat. Energy* **1**, 15029 (2016).
10. Griffith, K. J., Wiaderek, K. M., Cibin, G., Marbella, L. E. & Grey, C. P. Niobium tungsten oxides for high-rate lithium-ion energy storage. *Nature* **559**, 556-563 (2018).
11. Ko, M. *et al.* Scalable synthesis of silicon-nanolayer-embedded graphite for high-energy lithium-ion batteries. *Nat. Energy* **1**, 1 (2016).
12. Hassoun, J. *et al.* An Advanced Lithium-Ion Battery Based on a Graphene Anode and a Lithium Iron Phosphate Cathode. *Nano Lett.* **14**, 4901–4906 (2014).
13. Wang, B. *et al.* Ultrafast-charging silicon-based coral-like network anodes for lithium-ion batteries with high energy and power densities. *ACS Nano* **13**, 2307–2315 (2019).
14. Chae, C. *et al.* A High-Energy Li-Ion Battery Using a Silicon-Based Anode and a Nano-Structured Layered Composite Cathode. *Adv. Funct. Mater.* **24**, 3036–3042 (2014).
15. Zhang, L. *et al.* A Yolk–Shell Structured Silicon Anode with Superior Conductivity and High Tap Density for Full Lithium-Ion Batteries. *Angew. Chem. Int. Ed.* **58**, 8824–8828 (2019).
16. Ming, H. *et al.* High dispersion of TiO_2 nanocrystals within porous carbon improves lithium storage capacity and can be applied batteries to $LiNi_{0.5}Mn_{1.5}O_4$. *J. Mater. Chem. A.* **2**, 18938–18945 (2014).

17. Wang, C. *et al.* Fabrication and Shell Optimization of Synergistic TiO_2-MoO_3 Core–Shell Nanowire Array Anode for High Energy and Power Density Lithium-Ion Batteries. *Adv. Funct. Mater.* **25**, 3524–3533 (2015).
18. Aravindan, V. *et al.* A novel strategy to construct high performance lithium-ion cells using one dimensional electrospun nanofibers, electrodes and separators. *Nanoscale* **5**, 10636–10645 (2013).
19. Li, S. *et al.* High-rate aluminium yolk-shell nanoparticle anode for Li-ion battery with long cycle life and ultrahigh capacity. *Nat. Commun.* **6**, 7872 (2015).
20. Li, X. *et al.* Germanium Anode with Excellent Lithium Storage Performance in a Germanium/Lithium–Cobalt Oxide Lithium-Ion Battery. *ACS Nano* **9**, 1858–1867 (2015).
21. Brutti, S. *et al.* A high-power Sn–C/C–$LiFePO_4$ lithium-ion battery. *J. Power Sources* **217**, 72–76 (2012).
22. Chen, M. *et al.* High power and stable P-doped yolk-shell structured Si@C anode simultaneously enhancing conductivity and Li^+ diffusion kinetics. *Nano Res.* **14**, 1004–1011 (2021).
